# Supplementary material for: Are carbon emissions trading and green financial instruments synergistic? -Comprehensive quantitative research based on content analysis
Source: PLoS One. 2024 Mar 7;19(3):e0298601. doi: 10.1371/journal.pone.0298601 (PMC10919601; doi:10.1371/journal.pone.0298601)
Supplement: S1 Data — (ZIP) [file pone.0298601.s001.zip › Data/地级市Z_F工作报告.zip!/╣≤╓▌/┴∙┼╠╦«╩╨/└┤╫╘╣1⁄2╓┌║┼í╛┬φ┐╦╩2╛▌═°í┐_2010.docx]

各位代表：

　　现在，我代表市人民政府，向大会报告工作，请予审议，并请各位政协委员和列席的同志提出意见。

　　２００９年政府工作回顾

　　受国际金融危机的严重影响，２００９年是我市发展进程中非常艰难的一年。面对严峻考验，在省委、省政府和市委的坚强领导下，在市人大、市政协的监督、支持和帮助下，我们坚持以邓小平理论、“三个代表”重要思想为指导，深入贯彻落实科学发展观，切实按照“保增长、保民生、保稳定”的要求，努力克服困难，狠抓工作落实，较好地完成了年初确定的各项目标任务，呈现出经济回暖、结构优化、民生改善、社会稳定的良好态势。

　　全市生产总值完成４１２．３８亿元，增长１１．５％。全社会固定资产投资完成２４４．７８亿元，增长２３．８％。财政总收入和地方财政收入分别完成７４．０４亿元和３７．１２亿元，增长２０．５５％和２４．９４％，一般预算支出８２．１５亿元，增长３０．１７％。社会消费品零售总额完成１１０．８８亿元，增长１８．２％。城镇居民人均可支配收入和农民人均纯收入分别为１３１１６元和３０８３元，分别实际增长７．２％和１２．７％。年末金融机构存、贷款余额分别为４２４．０５亿元和３０８．２５亿元，分别比年初增加８２．１７亿元和８４．２６亿元。

　　一年来，我们着重做了以下工作：

　　第一，沉着应对国际金融危机带来的不利影响，经济形势回升向好。紧紧抓住中央扩内需、保增长的政策机遇，积极争取到四批中央新增投资７．９９亿元，一批多年来想办而又没有办成和久拖不决的重大项目获得国家批准并开工建设，为应对危机保增长提供了坚强支撑。面对４月份以来工业经济持续负增长、经济发展增速放缓的局面，以稳定煤炭、电力生产为核心，加强经济运行调节，从１１月份起工业经济转为正增长，为保持经济平稳较快发展奠定了坚实基础。认真落实国家“家电下乡”等扩大消费政策措施，全面推进“万村千乡”和“双百”市场工程建设，积极扩大农村居民消费，有效促进了经济增长。

　　第二，坚持以农民增收为核心，农业农村经济加快发展。认真落实强农惠农政策，市县投入农业专项资金１．７６亿元，增长２８．２２％，兑现农资综合补贴、种粮直补９５０４万元。加大“工业反哺农业、城市支持农村”力度，首批２２４户工矿企业与１６０个村结对，共投入各类资金２亿元。粮食生产连续７年实现丰收，总产量达８６．０２万吨，增长２．２１％。马铃薯、蔬菜、畜牧养殖等产业的规模化、集约化、商品化水平进一步提高，马铃薯种植面积达１８３万亩，产量达２２９万吨，较上年增长３０％；新增商品蔬菜基地２．１６万亩；肉类总产量达到１１．３４万吨，同比增长１８．４１％，畜牧业产值占农业总产值的比重为３８．６％，比上年提高１个百分点。农业产业化经营快速推进，土地流转加快。农民有组织进入市场的力度加大，新增农村专业合作社４９个。开展各类农民培训４４万余人（次）。建成沼气池２万口。

　　第三，加强基础设施建设，发展基础不断夯实。六沾铁路复线建设加快。长昆快速铁路、六盘水火车站改造及货场搬迁等工程前期工作进展顺利；公路建设取得重大突破，完成投资２２．８５亿元，其中市县财政投入４．３亿元，较上年增长３４．４％。水盘高速公路全面施工，六枝至镇宁高速公路开工建设，水城至六枝高速公路、杭瑞高速公路境内段前期工作加速推进。建成红果至响水、羊场至柏果等一批二级公路２９３公里，开工建设大河至木果、响水河至三块田等一批运煤公路３９７公里，建成通乡油路１８２公里，开工建设通村公路１０２０公里。动工建设六盘水月照机场试验段，机场路路基建设基本完成。开工建设中寨、野钟码头，建成毛口码头一期工程；黔中水利枢纽一期工程和白河沟供水工程开工建设，双桥、旧院、鱼洞坝、卡河水库前期工作有序推进。完成８．３７万亩烟水配套工程，治理病险水库６座，新增、改善和恢复有效灌溉面积４．５万亩，解决１３．４１万人农村饮水安全问题，治理水土流失面积１０７．８平方公里；开工建设１个５００ＫＶ和一批２２０ＫＶ、１１０ＫＶ输变电工程，完成投资１２．７亿元。加大农村电网改造力度，在全省率先实现户户通电；３Ｇ通信网络建设加快推进，移动、联通、电信等通讯公司投入信息基础设施建设３．６亿元；凉都大道全线建成通车，人民路东、西段及龙井北路建设有序推进，麒麟公园建成开园，市中心城区第二气源输配系统一期工程建成投入使用，水城河两岸截污管网全线贯通，建成市中心城区医疗垃圾集中处置场，城市功能进一步完善。

　　第四，加快推进工业结构调整，工业发展水平不断提高。进一步做大做强煤炭、电力、钢铁、建材等支柱产业。骨干煤矿建设继续加快，煤矿机械化采煤、瓦斯监控系统等装备水平进一步提高，全市原煤产量达５１２６．５８万吨，增长１４．５％；发耳电厂３＃机组建成，全市电力装机容量达６００万千瓦；盘县煤（焦、化）钢电一体化循环经济工业基地项目开工建设，水钢技改稳步推进，完成钢产量３１９．２１万吨，增长３．９％；盘县三合水泥厂等一批水泥生产项目建成，全市水泥年生产能力达５００万吨；煤化工发展加快，老鹰山煤基气化替代燃料项目一期工程完成设备安装，盘南煤化工、水城玉舍循环经济型煤焦化等一批重大项目前期工作加快推进；矿山机械、机电产品等产业生产能力进一步提升。有色金属工业、轻工业发展步伐加快。全市规模以上工业增加值完成１７５．２６亿元，增长４．８％。

　　第五，切实加强节能减排和生态环境建设，可持续发展能力进一步增强。对２３户年耗标准煤５０００吨以上的重点企业开展节能降耗监控。继续加大落后产能淘汰工作，重点对煤炭洗选行业进行整治，取缔关闭７７家洗煤厂。４个县级城镇污水处理厂建成投入试运行。积极推进煤层气综合利用，新增６．２５万千瓦瓦斯发电装机容量。省下达我市的节能减排目标基本完成。加强重点水域的保护和治理，玉舍水库库区环境整治取得初步效果，北盘江、三岔河出境断面水质达到水功能区Ⅲ类水质标准以上。加大生态建设和保护力度，完成石漠化综合治理５６．２平方公里、营造林面积１７．８５万亩，全市森林覆盖率达到３７％。继续推进国有重点煤矿采煤沉陷区综合治理。

　　第六，坚定不移地推进改革开放，经济发展活力不断增强。国有企业改革脱困工作有序推进，市水务有限公司组建完成，国有资本经营预算制度正式实施。金融改革步伐加快，邮政储蓄银行六盘水分行正式挂牌；投入４５００万元新增４１个农村金融服务网点，实现全市乡镇金融机构全覆盖。投融资体制改革取得新进展，运用ＢＴ、ＢＯＴ模式建设了一批基础设施。农村综合改革继续深化，集体林权制度主体改革基本完成。政府机构改革进展顺利。非公有制经济加快发展，实现增加值１５８．２４亿元，增长１２．５５％。与大连市及友好城市、周边地区的交往日益加深，区域经济合作不断深化。全年招商引资实际到位资金７４．５２亿元，同比增长２３．０４％。

　　第七，大力保障和改善民生，发展成果更多地惠及人民群众。各级财政共投入民生方面资金３４亿元，较上年增加了１１亿元。认真落实国家“五缓四减三补贴”等政策，全市就业形势稳定；市级财政投入１０００万元建立了创业基金，全市新增城镇就业１９２３７人，城镇登记失业率为４．１２％。全面启动实施城镇居民基本医疗保险；城镇职工基本养老、基本医疗、失业、工伤、生育等保险扩面征缴任务超额完成；发放城乡低保资金３．３亿元，共保障７．９万城镇困难人口和２８．８５万农村贫困人口，城乡低保实现了应保尽保、分类施保；新建１０所农村敬老院；成立了慈善总会；全市农村贫困家庭和城镇低保家庭的民用煤补助资金达１亿元以上；发放１４９８万元对１．１６万城乡困难群众实施了大病医疗救助。完成１４９７３户农村危房改造和１２万平方米的经济适用住房，发放廉租房租赁补贴１６６５万元，２．３万户煤矿棚户区改造前期工作进展顺利。加强扶贫开发工作，减少４．８万农村贫困人口。安全生产形势持续稳定好转，全年无重大以上事故，实现生产安全事故起数和死亡人数“双降”，全市煤矿百万吨死亡率为１．９３。信访和维稳工作进一步加强，确保了建国６０周年等重大节庆活动期间的社会稳定。扎实推进“平安六盘水”建设，群众安全感有所提高。

　　第八，加快发展社会事业，经济社会发展的协调性不断增强。各级财政用于教育事业的投入进一步加大，共安排教育资金１０亿元，较上年增加了３亿元。教育基础设施不断改善，完成农村义务教育阶段薄弱学校改造工程７０所；“两基”巩固提高工作取得成效，六枝特区、水城县通过省政府“两基”复查和“普实”验收；市一中双水校区和市三中凤凰校区建成招生；整合地企职业技术教育资源取得实质性进展；高等教育实现重大突破，六盘水师范高等专科学校升格为六盘水师范学院；开工建设六盘水职业技术学院二期工程。启动医药卫生体制改革；城乡医疗条件进一步改善，市人民医院住院综合大楼主体工程基本完工，新建和改扩建１４个乡镇卫生院、３个社区卫生服务中心和３１９所村卫生室；新型农村合作医疗参合率达９７．４５％；认真做好甲型Ｈ１Ｎ１流感防控工作；完成７．５万户地氟病防治炉灶改良任务；理顺了市级医疗机构管理体制。人口计生工作水平稳步提高，人口出生率为１２．６％。，符合政策生育率达９５．１２％。科技、文化、体育、广播电视等社会事业继续发展。汽车应用压缩焦炉气（ＣＯＧ）技术取得成功并通过省级鉴定；完成１３个乡镇综合文化站建设，保护了一批文物和非物质文化遗产；我市培养输送的运动员在第十一届全国运动会上取得一金一银好成绩；建成８５５个２０户以上自然村广播电视“村村通”工程，完成市中心城区数字电视整体转换平移工作。老龄、妇女儿童和残疾人事业取得新进步。国防建设进一步加强。民族、宗教、旅游、外事、侨务、对台、档案、气象、人防、青少年、工会、红十字等工作取得新进展。

　　过去一年，我们切实加强民主法制建设和政府自身建设。自觉接受人大的法律监督、工作监督和政协的民主监督。认真办理人大代表建议和政协提案。各类公开办事制度不断完善，政府决策科学化、民主化水平不断提高。“五五”普法依法治理工作深入开展，依法治市全面推进。扎实开展深入学习实践科学发展观活动。继续推进惩治和预防腐败体系建设，切实加强行政监察和审计工作。

　　各位代表！在历史罕见的挑战和风险面前，过去一年取得的成绩来之不易。成绩的取得，是党中央、国务院和省委、省政府总揽全局、审时度势、坚强领导的结果，是市委深入研判形势、科学决策、有力应对的结果，是全市各族干部群众凝聚共识、迎难而上、团结拼搏的结果。在此，我代表市人民政府，向全市工人、农民、知识分子、干部，向各民主党派、工商联、无党派人士、人民团体，向驻市人民解放军指战员、武警部队官兵、公安政法干警，向所有关心和支持我市改革发展的各界人士，表示衷心的感谢并致以崇高的敬意！

　　在充分肯定成绩的同时，我们也清醒地看到，经济社会发展中还存在不少困难和问题，主要是：资源依赖型经济结构和粗放型发展方式尚未根本改变，资源综合利用水平不高，节能减排压力较大；城乡居民收入差距大，统筹城乡发展任务十分艰巨；经济与社会发展协调性还不够；政府自身建设还存在薄弱环节。对于这些问题，我们将高度重视、认真对待，采取有力措施加以解决。

　　２０１０年政府工作主要任务

　　今年是全面完成“十一五”规划、衔接“十二五”时期发展的关键之年，也是经济形势复杂多变的一年，总体上机遇与挑战同在、希望与困难并存。政府工作的总体要求是：以邓小平理论和“三个代表”重要思想为指导，深入贯彻落实科学发展观，全面贯彻党的十七大和十七届三中、四中全会及中央、省经济工作会议精神，坚持“能快则快、又好又快”，以发展循环经济为统领，以扩大投资为抓手，以调整经济结构和转变发展方式为主线，以深化改革扩大开放为动力，以保障和改善民生为根本出发点和落脚点，以维护社会和谐稳定为保障，统筹做好各项工作，确保全市经济社会又好又快发展，圆满完成“十一五”规划目标，为“十二五”时期发展打下良好基础。

　　全市经济社会发展的主要预期目标是：生产总值增长１２％；全社会固定资产投资增长２０％；财政一般预算收入增长１６％以上；社会消费品零售总额增长１６％以上；城镇居民人均可支配收入增长６％，农民人均纯收入增长８％；城镇登记失业率控制在４．５％以内；人口出生率控制在１２．８５％。以内；完成省下达的节能减排指标。

　　做好今年政府工作，我们将把握好以下原则：一是坚持扩大投资与扩大消费相结合。在继续推进重大项目建设、着力扩大投资规模、优化投资结构的同时，认真落实国家扩大消费需求的各项政策措施，不断增强群众消费能力，充分发挥消费对经济的拉动作用。二是坚持把当前发展与长远发展相结合。在保持经济平稳较快增长的同时，着眼长远，多做打基础、利长远的工作，把改善基础设施条件、调整经济结构和加快发展方式转变作为贯彻落实科学发展观的重要目标和战略举措，在发展中促转变，在转变中谋发展，为实现更高水平、更持久的发展创造条件。三是坚持加速工业化、加快城镇化与推进农业现代化相结合。把工业化作为壮大经济实力、提高生产水平的核心任务，把提高城镇化率作为统筹城乡发展、有效扩大内需的重要抓手，把农业现代化作为促进农民增收、建设社会主义新农村的根本途径，使之相互促进、协同发展。四是坚持加快发展与改善民生相结合，把加快发展作为解决民生问题的根本途径，把改善民生作为加快发展的出发点和落脚点，把公共资源配置更多地向民生领域倾斜，在加快发展中着力改善民生，在改善民生中实现科学发展。

　　围绕上述总体要求、主要预期目标和主要原则，我们将着力做好以下八个方面的工作。

　　（一）大力发展循环经济，努力推动经济发展方式的转变

　　紧紧抓住国务院将我市纳入资源富集区开展循环经济区试点这一难得的重大历史机遇，把加快发展循环经济作为转变经济发展方式的重要抓手，以循环经济理念布局全市经济发展的推进方向、重点领域和重大项目，唱响循环经济发展的主旋律。

　　切实抓好循环经济总体规划编制工作。按照“减量化、再利用、资源化”的要求，以推进资源节约、资源综合利用和清洁生产为重点，加强循环经济发展专题研究，制定全市循环经济发展总体规划及煤炭、电力、钢铁、建材、化工等重点产业专项规划。在此基础上，确定年度推进计划，并建立循环经济项目库。同时做好与国家主体功能区规划的衔接工作。

　　深入开展循环经济试点示范。一是抓好企业层面的小循环。大力支持和鼓励境内企业积极发展循环经济，改进设备和工艺流程，推行清洁生产。重点在废渣、废水、废气、余热、余压的综合利用上下功夫，加快推进煤矸石发电、煤矸石制砖、粉煤灰生产建材及洗煤废水、矿井水的循环利用和煤矿瓦斯抽采、焦炉煤气的回收利用，积极推广尾矿回填充填新技术，培育一批循环经济试点示范企业；二是抓好集中区层面的中循环。选择一些特色产业集聚区或工业集中区开展循环经济试点，总结经验，推广实施。实行区内区外差别政策，引导区外企业向集中区搬迁集中，实现集中区土地集约使用、废物集中处理，搞好关联企业之间的资源链、加工链和产品链的对接延伸，培育一批生态型循环经济集中区或基地。建立健全组织机构，充分运用六盘水市城市总体规划第三轮修编成果，重点抓好红山石桥、老鹰山、红果以及发耳、黑塘、大用、木岗、盘东北、盘南等循环经济新型工业集中区建设；三是抓好社会层面的大循环。大力开展循环经济的知识普及和宣传教育，倡导健康、文明、节俭、适度的消费理念，提高全民发展循环经济的意识和公众参与度。

　　建立促进循环经济发展的鼓励政策。在积极争取国家政策支持的同时，认真清理和梳理循环经济发展的有关优惠政策和办事程序，并完善地方配套政策，形成促进循环经济发展的政策体系。切实落实好国家节约资源、开发绿色能源、可再生资源利用等优惠政策，积极利用清洁发展机制等国际资本、各种社会资金参与循环经济项目建设，促进循环经济产业发展。

　　坚持把发展循环经济同节能减排工作有机结合起来，通过发展循环经济实现节能减排，通过节能减排促进循环经济发展。一是抓好结构性节能减排。严格执行国家产业政策和行业准入标准，加快淘汰高耗能、高排放、低效益的落后生产力。加强固定资产投资项目节能评估和审查；二是抓好工程性节能减排。深入实施建筑节能、绿色照明、电机改造等“十大重点节能工程”。鼓励企业采用新技术、新工艺、新设备实施节能减排重大技术改造，努力实现能耗稳定下降和污染物稳定达标排放；三是抓好管理性节能减排。着力抓好电力、冶金、建材等重点领域、重点行业和年耗能５０００吨标准煤以上企业的节能降耗监控。切实抓好污水处理厂、医疗垃圾处理场、垃圾填埋场和火电厂脱硫设施的运行管理。

　　在大力抓好循环经济和节能减排的同时，进一步加强生态环境建设和资源集约节约利用。把石漠化治理作为生态建设的首要任务，扎实推进４个县、特区、区石漠化综合治理，完成７８．７平方公里治理任务。继续推进林业重点工程和巩固退耕还林成果项目建设，完成营造林任务１５万亩以上。加强森林防火工作，森林火灾受害率控制在０．６７％。以内。加强玉舍水库等城镇和农村集中式饮用水源地保护与治理。加强地质灾害防治。全面完成国有重点煤矿采煤沉陷区综合治理。加强乌蒙山国家地质公园等自然遗产保护，并积极做好申报世界自然遗产的有关工作；坚持保障发展与保护资源并重，提高土地、矿产、水等资源的集约和节约利用水平。实施新一轮土地利用总体规划，重新划定永久性基本农田保护区。开展城乡用地增减挂钩工作，努力保障城市发展、产业发展和基础设施建设用地需求。切实落实国家和省关于加大资源综合利用的政策，进一步完善资源有偿使用制度。

　　（二）坚持以农民增收为纲，加快发展现代农业

　　切实按照“稳粮保供给、增收惠民生、统筹促发展、强基增后劲”的要求，把统筹城乡发展作为全面建设小康社会的根本要求，把增加农民收入作为农业农村工作的重中之重，把发展现代农业、提升农民素质作为全面推进农业跨越式发展的重要任务，把建设社会主义新农村作为农村经济社会全面发展的强大动力，充分利用我市立体气候和生物多样性突出、可开发利用山地坡地较多、农民增收空间较大、特色农业发展前景广阔的优势和潜力，围绕“大种植、大养殖、大加工、大市场”目标，以“农业产业化、农业标准化、生产要素社会化、土地流转规范化”为抓手，努力促进我市农业的跨越式发展。

　　农业产业化方面，因地制宜发展特色农产品，着力扩大农产品规模、改善品质、提高商品率。在稳定发展粮食生产、确保粮食增产２％以上的同时，大力发展畜牧养殖、马铃薯、蔬菜、干鲜果、茶叶、苦荞、生姜、中药材、烤烟等特色产业，完成马铃薯种植面积２００万亩，建成３０万亩马铃薯商品基地；新增１万亩商品蔬菜基地；实现畜牧业产值占农业总产值的比重提高１个百分点以上。切实加大对农业产业化龙头企业的政策、资金扶持力度，大力发展农村专业合作社。农业标准化方面，坚持以工业生产模式经营农业，把选育良种、种植养殖、贮存保鲜、加工销售等各个环节纳入标准化管理轨道。选择一些特色农产品或主导产业，制定无公害农产品的地方标准及生产技术操作规程，逐步提升农产品质量安全水平。积极推进农业机械化。生产要素社会化方面，切实调动全社会的资源和力量，引导和鼓励城市、企业、民间的技术、资金、人才等生产要素向农业和农村流转；研究制定“统筹城乡发展和工业反哺农业、城市支持农村”的政策措施，鼓励各种社会力量开展乡村结对帮扶，参与农业产业发展。土地流转规范化方面，把推动土地流转作为农业发展的重要环节，在确保不改变土地用途的前提下，按照“依法、自愿、有偿、规范”的原则，加强管理和服务，发展多种形式的适度规模经营，促进土地流转。

　　认真落实各项强农惠农政策，确保各项涉农补贴资金及时足额发放到农民手中。进一步加大市县财政对“三农”投入。提高农村金融服务质量和水平，引导更多信贷资金投向“三农”，切实解决农村融资难的问题。加强农民适用技术和转移就业培训，大力发展劳务经济，引导和鼓励农村富余劳动力向二、三产业和城镇转移。加强农村水电路气等基础设施及农村“五改”工程建设，在交通沿线、城镇周围、工矿附近建设一批具有地方特色和现代化气息的民居，不断改善农村生产生活条件和乡村面貌。加强农村基层组织建设，选好致富带头人。

　　（三）坚定不移地推进工业化，不断提升经济综合实力

　　以发展现代工业为方向，加快推进工业优化升级。按照“数量扩张与质量提升并重”的方针，改造提升煤炭、电力、钢铁、建材等传统产业。以建设现代化矿井为目标，科学开发、高效利用煤炭资源，做大做强国有煤炭企业，加快中小型煤矿资源整合和技术改造，积极推进大矿帮扶小矿和小煤矿托管，加大对地方煤矿整合技改新建矿井的支持力度，进一步提高煤炭产业集中度和采煤机械化程度。重点开工建设化乐、黑拉嘎、发耳二矿、响水煤矿二期、玛依西、玛依东等大中型煤矿，力争全市原煤产量达６０００万吨。继续抓好煤炭洗选行业治理整顿，加快建设一批大型高效环保洗煤厂。加强煤电油运等经济运行调节，保持工业生产稳定；开工建设六枝电厂、水矿汪家寨矸石电厂、盘县电厂“上大压小”、四格坡上牧场风力发电等一批电源点项目；围绕建成西南地区长材精品基地的目标，大力支持水钢５００万吨钢配套项目和盘县煤（焦、化）钢电一体化循环经济工业基地建设，推进高技术含量、高附加值产品研发；加快建材产业扩能提质步伐，建成一批新型水泥生产线和建材厂。

　　按照“延长、配套、接续”的路径，培育壮大新兴产业。加快发展现代煤化工，延长产品增值链。力争开工老鹰山煤基气化替代燃料项目二期工程，加快推进盘县红果、水城玉舍循环经济型煤焦化和盘南煤化工等一批新型煤化工项目建设。有序关闭淘汰一批不符合产业政策的焦化企业；以矿山机械机电制造、加工为重点，加快煤矿井下综合采掘、提升、运输设备和洗选设备等产品的研发、生产和市场开拓，努力形成在全省乃至西南地区具有一定影响力的品牌产品和装备制造业产业群；高起点、高质量发展电解铝等有色金属工业，着力搞好精深加工，延长电力产业链，力争把六盘水建成重要的电解铝、铝加工基地；加快发展食品加工、农副产品加工、民族制药等产业，不断提高轻工业占工业经济的比重。

　　按照“市场导向、发挥优势、重点突破”的思路，加大承接东部地区产业转移力度，力争将我市列为国家承接东部产业转移的试点城市。充分依托产业优势，主动承接关联配套产业转移；依托资源优势，主动承接深加工产业转移；依托成本优势，主动承接劳动密集型产业转移；依托区位优势，主动承接加工贸易和服务业产业转移。在做好资源依托型产业转移的同时，积极承接非资源型产业，避免出现产业结构单一、单纯依靠资源发展的局面。

　　（四）加快以交通、水利、电网和信息为重点的基础设施建设，不断改善加快发展的基础条件

　　以构建现代综合运输体系为目标，加快推进铁路、公路、机场、航运等交通基础设施建设。继续配合抓好六沾铁路复线建设；实施六盘水火车站站房改造、货场搬迁工程；加快推进水红铁路预留站和铁路战略装车点的建设；争取开工建设长昆快速铁路境内段；积极做好水红铁路复线、毕水兴快速铁路、织金－水城－攀枝花城际铁路前期工作。强力推进水盘高速公路、六枝至镇宁高速公路建设；开工建设水城至六枝高速公路和杭瑞高速公路境内段，做好市境内“省高规划”其他路段及市中心城区外环高速公路前期工作；全面建成玉舍至马场桥公路，大力推进响水至瓦窑田、羊场经英武至大山二级公路建设；开工建设第四批运煤公路１００公里；加快推进姬关营至龙场、鸡场至龙场、顺场至龙场等通乡油路建设；加强农村公路管理养护。推进六盘水月照机场建设。建成中寨、野钟水运码头。高度重视交通干线沿线的产业发展规划和布局。

　　以构建城乡水资源保障体系为目标，加快推进水利基础设施建设。配合抓好黔中水利枢纽一期工程建设，加快推进白河沟供水工程建设，开工建设双桥、渔洞坝水库，继续推进旧院、卡河水库前期工作，启动开展英武水库前期工作。积极争取实施六盘水灌区续建配套和节水改造、六枝中型灌区节水配套改造项目。大力实施农村饮水安全工程，新增解决５万人农村饮水安全问题。完成４．８３万亩烟水配套工程。新增、改善和恢复有效灌溉面积４．５万亩。治理病险水库４座。治理水土流失面积１３平方公里。大力实施以管代渠、长距离管道送水。加强对全市地下水资源的勘探，编制地下水开发利用规划。进一步完善城市供水管网，有序关闭市中心城区地下水开采。加强水利工程管理，促进水利工程持久发挥作用。

　　以构建生产生活用电保障体系为目标，加快推进城乡电网建设。开工建设５００ＫＶ六盘水输变电二期工程和３个２２０ＫＶ、１６个１１０ＫＶ输变电工程，力争完成投资２０亿元以上。加大城市电网改造力度。进一步完善农村电网。着力完善地方煤矿双回路供电设施。积极争取国家和省的支持，推进用电大户直供电和自备电厂供电。

　　以构建现代信息网络体系为目标，加快推进信息基础设施建设。坚持共建共享和以需求促应用的原则，围绕打造“数字六盘水”，全力推进３Ｇ通信网络的建设与发展，开展全市数据处理交换中心、数据采集系统、基础数据库建设前期工作；积极推进电信网、互联网和数字电视网的“三网融合”以及各类信息平台的整合；深化信息技术在电子政务、电子商务、城市管理、社会治安综合治理、远程教育等行业和领域的应用，推进信息技术向农村延伸。

　　（五）加快推进城镇化进程，努力提高城镇发展水平

　　按照“完善城市功能、改善人居环境、规范城市管理、发展城市经济”的要求，着力加强城乡规划、建设、管理和城市产业支撑。

　　科学定位，高起点规划城市。切实发挥规划对城市产业布局、综合交通、土地利用、生态环境、历史文化保护、城镇特色塑造等重大问题的指导作用，加快形成具有六盘水特色的城镇体系。围绕打造“峰丛景观———山水园林城市”和“中国凉都”品牌，发挥城市特色，搞好城市设计，美化城市景观，提高城市品质，进一步做好市中心城区的控制性详细规划和修建性详细规划，同时结合工业集中区建设和新区拓展，着力解决城市南北过窄问题。高标准、高质量修编和实施平寨、红果两个次中心城市的规划。加快编制重点小城镇、工矿区和乡村的规划。认真执行城乡规划法，加大依法打击“两违”建筑力度，维护规划的严肃性。

　　创新发展方式，高标准建设城市。一是推进基础设施配套化。以城市路网和污水垃圾处理系统建设为重点，统筹考虑水、电、气、通信、绿化等城市基础设施和学校、医院等配套设施建设，扩大城市规模，完善城市功能，提升城市品位，不断增强城市综合承载能力。重点抓好凤凰山片区建设、水城河综合治理和老城改造；全面建成人民路东、西段；启动实施花园路北段工程建设，推进水钢南出口不畅问题的解决。加快完善城市公交基础设施。推进凉都体育中心建设。二是推进投资主体多元化。除公益性较强的公用事业坚持由政府主导或控股外，坚决放开一般性竞争领域，激活社会资本参与城市建设、经营和管理。加大商业设施建设力度，吸引社会资本投入，带动城市财富积累，形成城市投资、建设和发展的良性循环。三是推进土地供应规范化。按照先规划、再收储、后出让的程序，强化土地储备，做好征地拆迁安置工作，推进净地出让。四是推进城市建设风格精品化。着重提高城市绿化、道路、雕塑、楼宇的设计水平，形成风格和色彩协调统一的形象，突出城市特色和魅力。

　　创新管理手段，科学管理城市。坚持建管并重、重在管理的原则，实施科学化、精细化、人性化、柔性化城市管理，提升城市管理水平。加强社区标准化建设，推进城市管理重心下移，强化“门前三包”工作；加强宣传教育，提高市民素质，增强市民参与城市管理的主动性和积极性；深入开展“整脏治乱”工作；积极推行市场化卫生保洁机制；实行严管重罚，严厉处罚违反城市管理规定的行为；下大力解决市中心城区道路交通拥堵问题。

　　加快发展服务业，繁荣城市经济。一是围绕打造“凉都”品牌，积极发展旅游产业。加快开发北盘江大峡谷等重点景区景点，积极抓好以城郊农家乐为主的乡村旅游，加快建成城郊旅游休闲经济带。二是大力发展现代物流业。用现代信息技术改造提升物流业，降低全社会物流成本，缩短物流周期，加快建设区域物流中心，争取将我市列入全国物流试点城市。积极规划并启动建设一批大型物流园区。治理整顿市中心城区批发市场。三是加快发展房地产业。鼓励引导发展老年公寓、酒店式公寓等多元化房地产市场。强化房地产市场管理，规范房地产市场秩序。用好、用足、用活住房公积金。四是大力发展金融保险、邮政通信、交通运输、商贸流通、餐饮娱乐等产业，积极发展文化、体育、卫生、广播电视等产业。在抓好城市产业发展同时，认真贯彻落实国家和省促进消费的政策措施，有效扩大消费需求，积极调整经济增长的动力结构。继续实施国家“家电下乡”等政策，扎实推进“万村千乡”和“双百”市场工程，扩大农村居民消费。加强市场监管，努力营造便利、安全、放心的消费环境。

　　推进人口转移，加快城镇化进程。认真落实促进农村人口向城市转移户籍制度改革政策，解决好农民工就业、居住、就医、子女就学等方面的困难和问题，带动有条件的农民工特别是新生代农民工转变为城市居民，推动农村人口向城市合理有序转移，促进城镇化率的提高。

　　（六）继续深化改革开放，加快发展非公有制经济

　　坚持市场原则，加快国有企业产权多元化改革，优化企业股权结构，完善国有企业经营预算制度，稳步推进国有企业改革脱困工作，增强国有经济活力、控制力和影响力。按照“有进有退、优进劣退、有序进退、合理流动”的要求，进一步健全国有资产管理体系，加强国有资产监管，确保国有资产保值增值。加快供销合作社改革发展。继续深化农村综合改革，扎实推进集体林权制度配套改革，开展农村集体建设用地流转试点，启动宅基地确权登记发证工作。推进财税体制改革，强化财政刚性预算，科学、规范地加快财政支出，切实盘活各类沉淀资金，彻底清理“小金库”。推进投融资体制改革，继续把扩大投资规模作为推动我市经济又好又快发展的基础性工作，认真解决项目建设中土地、环保、资金、施工组织、建设环境等问题，确保在建、续建项目的建设进度；强化项目前期工作，争取更多新建项目开工建设。切实做好“十二五”规划的编制工作。做大做强城市商业银行。做好农村信用社改制工作。建立健全政、银、企合作协调机制，加大信贷支持力度。支持、规范发展小额贷款公司。加快培育和规范发展各类民间商会、行业协会及金融服务中介机构和产权交易市场。做大做强投融资平台，提高政策性担保机构的融资担保能力。鼓励支持符合产业政策、成长性好、有上市意愿的企业上市或发行企业债券。进一步深化行政体制改革，全面完成市、县政府机构改革，重塑和优化工作流程，提高行政效能。

　　进一步扩大开放。认真学习借鉴外地先进经验，开阔眼界，拓宽思路，加快发展。认真贯彻落实市委最近出台的《关于全面加强机关效能建设进一步优化经济社会发展软环境的实施意见》等“１＋８文件”精神，切实改善投资软环境，加大招商引资力度。推进区域经济合作与联动，加强与泛珠三角、东盟等经济区以及周边地区、友好城市的联系和往来，更好地在区域经济中履行职责和发挥作用，开拓产品的国际国内市场。

　　大力推进非公有制经济又好又快发展。坚持“非禁即允”和平等待遇的原则，凡是国家法律法规未明令禁入的行业和领域，非公有制经济都可以进入；切实帮助非公有制企业解决发展中资金、人才、技术、土地、管理等要素短缺的问题，指导非公有制企业建立健全现代企业制度，提升企业素质；做好项目和信息服务，引导非公有制企业结合我市产业结构调整，积极进入轻工业、装备制造业、农产品加工业等重点领域发展。

　　（七）大力发展各项社会事业，加快推进公共服务均等化

　　努力办好人民满意的教育。加大教育投入，改善办学条件，提高教育质量，促进义务教育均衡发展、高中教育扩大发展、职业教育跨越发展、高等教育加快发展，推进教育公平进程。巩固提升“两基”成果，继续实施中小学校舍安全工程和农村义务教育阶段薄弱学校改造工程，完善农村义务教育经费保障机制，切实解决家庭困难学生、农民工子女平等接受教育问题。推进市中心城区教育资源整合，努力解决教育资源短缺特别是大班额问题。加快推进“普及高中阶段教育”计划的实施。抓住国家大力发展职业教育机遇，发展一批中等职业学校，力争到２０１１年，未能升入普通高中的初中毕业生，都能接受中等职业技术教育。以建成西南地区重要的“职业技术教育培训基地”和“技能型人才输出基地”为目标，加快推进地方和企业的职业技术教育资源整合。加快六盘水职业技术学院二期工程建设。加强六盘水师范学院软硬件建设，切实做好迎接国家对本科院校办学条件进行评估的准备工作，并积极为申办“贵州理工学院”创造条件。加强民族教育，规范发展民办教育。加强教师队伍建设，强化师德师风，全面提升教学教育质量。

　　努力提高人民健康水平。坚持公共医疗卫生的公益性质，着力满足人民群众就医需求。完善公共卫生服务体系，加大重大传染病的防控，积极应对重大突发公共卫生事件。加强医药价格监管，切实减轻群众基本用药经济负担。进一步改善城乡医疗卫生条件，建成投入使用市人民医院住院综合大楼并做好门诊大楼开工建设的前期工作，启动实施市妇女儿童医院改扩建工程，加快水钢医院搬迁步伐；新建和改扩建一批乡镇卫生院、社区卫生服务中心、村级卫生室，加强基层医疗卫生人员培训。积极筹建市中医院，扶持中医药事业发展。进一步规范和整顿各级医疗机构。加强医德医风建设。加大统筹解决人口问题的力度，夯实基层基础，继续抓好流动人口管理，综合治理出生人口性别比失衡问题，稳定低生育水平。

　　努力满足人民精神文化需求。深化文化体制改革，完善城乡公共文化服务体系，加快推进县级公共图书馆和文化馆、乡镇综合文化站、社区文化活动室的建设改造或设备配置工作。切实推进影城院线建设。加强文化遗产保护和合理开发，积极申报联合国教科文卫组织“人类非物质文化遗产代表作名录和国家级文化遗产保护项目”。继续办好凉都消夏文化节。完成７０３３个２０户以上自然村广播电视“村村通”工程；全面整合有线电视网络资源，完成六枝特区、盘县数字电视整体平移工作并向有条件的乡村延伸。强化国防建设，深入开展“双拥”创建活动。推进老龄、妇女儿童和残疾人事业的发展。加强精神文明建设，促进社会风气进一步好转。继续做好科技、体育、民族、宗教、侨务、对台、档案、地方志、青少年、工会、红十字等工作。

　　（八）大力保障和改善民生，切实解决人民群众最关心、最直接、最现实的利益问题

　　扎实做好就业和社会保障工作。实施更加积极的就业政策。继续对困难企业实行“五缓四减三补贴”政策，加强对农民工和下岗失业人员的技能培训，千方百计稳定和扩大就业。壮大创业基金规模，大力实施以创业带动就业工程，力争纳入省级创业试点城市。努力开发公益性岗位帮助困难就业对象实现就业，确保零就业家庭动态清零。统筹做好高校毕业生、复员退伍军人和城镇新增就业人员的就业工作。确保全市城镇新增就业２万人。以“全民社保”为目标，加快完善社会保障体系。继续扩大城镇职工基本养老、基本医疗、失业、工伤、生育等保险覆盖面，力争参保人数分别达６万人、５８．１４万人、８．６万人、１７万人和５万人。启动钟山区新型农村社会养老保险试点工作。认真做好被征地农民的基本养老保障工作。全面推进城镇居民基本医疗保险，积极探索建立城镇职工基本医疗保险和生育保险市级统筹办法，稳步提高新型农村合作医疗参合率。加强城乡低保工作，切实做到动态管理下的应保尽保、分类施保。新建１０个农村敬老院。大力发展以扶老、助残、救孤、济困为重点的社会福利和慈善事业。加强扶贫开发工作，减少农村贫困人口５万人。实施“温暖工程”，启动市中心城区集中供暖工程前期工作，继续做好农村民用煤供应和城市低收入家庭民用煤补贴工作。切实抓好保障性安居工程建设。完成省下达的农村危房改造任务。采取租售并举等办法，搞好廉租住房建设，规范和加强经济适用住房建设，不断提高人均住房面积１５平方米以下的城镇低收入家庭住房保障水平。启动２．３万户煤矿棚户区改造。在市中心城区和县城启动农民工公寓建设。

　　扎实做好安全生产工作。牢固树立“以人为本、安全发展”的理念，严格落实安全生产责任制，强化安全生产基层基础工作。正确处理好安全与生产的关系，切实做到不安全不生产、抓安全促生产。重点抓好煤矿、非煤矿山和道路交通等重点领域安全专项整治工作，加强安全隐患排查治理，坚决遏制重特大事故的发生。加强食品药品质量监管和市场监管，保障人民群众的健康安全。

　　扎实做好社会稳定工作。坚持发展是硬道理、稳定是硬任务，切实增进团结、维护稳定。加强信访工作，大力开展信访积案清理化解，通过“化积案”来“化积怨”；积极开展矛盾和纠纷的排查、调处，努力使矛盾化解于未萌、群体性事件化解于无形。切实加强对“虚拟社会”的管理，建立日常舆情分析机制和突发事件的新闻发布快速反应机制，防止“虚拟社会”对现实社会的不利影响和放大效应。加强应急管理工作，不断提高应对突发事件的快速反应和有效处置能力。全面完成“五五”普法。广泛开展“平安六盘水”创建活动，深入开展“打黑除恶”等严打整治专项行动，依法防范和打击各种违法犯罪活动，努力营造和谐安定的社会环境。

　　加强政府自身建设

　　做好今年政府的各项工作，必须进一步加强政府自身建设，努力提高“五种能力”，切实增强广大干部的执行力，确保各项决策部署不折不扣地落到实处。

　　第一，加强学习、提升素质，不断提高政府执政能力。切实把加强学习作为一项重要任务来抓。通过学习，促进领导干部在思想上、政治上与党中央保持高度一致，正确把握上级动态，超前谋划，积极应对，不断提高执政能力；促进领导干部以系统的思维和统筹的办法谋划推动发展，切实解决实际工作中的重点、难点问题，实现科学决策、科学施政、科学管理。

　　第二，解放思想、与时俱进，不断提高政府创新能力。坚持把解放思想置于万事之先，教育引导各级干部从不合时宜的思想观念中解放出来、从固有的思维模式中解放出来，用创新的思维和措施来解决发展中的困难和问题。只要符合科学发展观、符合六盘水的实际、符合人民群众的根本利益，就要大胆闯、大胆干。坚决反对为不干事找借口、为干不成事找理由。

　　第三，转变职能、改进作风，不断提高政府服务能力。牢固树立服务为民的理念，带着深厚的感情为人民谋福祉，使改革发展成果更多地惠及人民群众。强化行政效能建设，坚决克服说而不做、议而不决、决而不行、抓而不实的不良作风。加强行政问责，切实解决好行政“不作为、慢作为、乱作为”现象。强化政府督查工作，确保政令畅通。从严管理干部，对违反党性原则的人和事不留情面，对工作不力和作风不正的干部不姑息迁就，对出了问题的干部不包庇袒护。

　　第四，依法行政、强化监督，不断提高政府依法治市能力。强化依法行政意识，提高依法行政能力，依靠法治化解社会矛盾、协调社会利益关系，保障社会公正和谐；依靠法治加强社会管理，加强法律援助和人民调解工作，保障社会安定有序；依靠法治深入整顿和规范市场经济秩序，保障经济平稳较快发展。自觉接受人大及其常委会的法律监督、工作监督和政协的民主监督，广泛听取民主党派、工商联、无党派人士和各人民团体的意见。认真接受新闻舆论和人民群众监督，集中各方智慧和力量共同做好政府工作。继续加强行政监察和审计监督。

　　第五，廉洁自律、率先垂范，不断提高政府勤政廉政能力。认真执行党风廉政建设责任制，落实“一岗双责”。扩大政务公开范围，以公开促公正，以开明保廉明，增强行政透明度，提高政府公信力。认真落实领导干部廉洁从政的各项规定，努力做到自身正、自身净、自身硬。树立艰苦奋斗、勤俭创业的思想，坚决反对讲排场、摆阔气，反对挥霍奢侈、铺张浪费，以廉洁的政府形象取信于民。

　　各位代表！做好今年各项工作，任务艰巨，意义重大。让我们紧密团结在以胡锦涛同志为总书记的党中央周围，以更加坚定不移的信念、求真务实的干劲、昂扬向上的风貌，攻坚克难，锐意进取，齐心协力，扎实工作，奋力开创全市经济社会发展新局面！
